# Modular protein frameworks via supramolecular synthons

**DOI:** 10.1007/s12551-025-01323-9

**Published:** 2025-06-09

**Authors:** Niamh M. Mockler, Peter B. Crowley

**Affiliations:** School of Biological and Chemical Sciences, University of Galway, University Road, Galway, H91 TK33 Ireland

**Keywords:** Affinity tag, Crystal engineering, Macrocycle, Protein assembly, Recognition

## Abstract

Controlled protein assembly and protein crystal engineering are routes to new types of biomaterials. In this review, we examine how crystal engineering concepts, including polymorph searching, molecular tectonics and supramolecular synthons, can be adapted and applied in protein-based systems. We explore ‘mix-and-match’ approaches, as established with modular frameworks that combine interchangeable components. We review the numerous current methodologies in protein assembly and crystal engineering from (de novo) designed proteins to metal-mediated or ligand-mediated strategies. Commercially available synthetic receptors such as macrocycles are useful protein assembly mediators and are advantageous in their applicability to diverse protein targets. We highlight the use of calixarenes, cucurbiturils and proteins as building blocks (tectons), showing that reproducible inter-tecton structural units (synthons) have applications in directing protein assembly and crystal engineering.

## Introduction

Porous materials have uses in storage, separation, sensing and catalysis (Furukawa et al. 2013; Lin et al. 2019; Feng et al. 2019; Geng et al. 2020; Little et al. 2020). Functional, biocompatible, biodegradable and sustainable, porous protein crystals are of special interest to the biophysicist, the chemist and the industrialist. The solvent channels (pores) of protein crystals can accommodate guests, from small ions to macromolecules. For instance, naturally occurring crystals of the cypovirus polyhedrin protein (polyhedra) encapsulate virus particles, protecting them from harsh physico-chemical conditions (Coulibaly et al. 2007). In semi-synthetic systems, porous protein crystals have been used (1) for storing exogenous materials such as nanoparticles and enzymes and (2) as catalysts or reaction vessels (Margolin and Navia 2001; Abe et al. 2015; Kowalski et al. 2016; Uchida et al. 2018; Heater et al. 2020; Nguyen et al. 2021; Kojima et al. 2022; Edwardson et al. 2022). Protein crystals can also serve as scaffolds for the structure determination of small molecules, peptides or other proteins (Ni and Tezcan 2010; Huber et al. 2018; Maita 2018; Ernst et al. 2019; Matsumoto et al. 2019; Kojima et al. 2023). About half of the protein crystal structures in the Protein Data Bank are reported to have ≤ 50% solvent content. Highly porous crystals, on the other hand, with solvent contents exceeding 80%, represent < 5% of the deposited structures (Kowalski et al. 2016; Rennie et al. 2018; Li et al. 2023). Strategies for producing (highly) porous protein crystals may be beneficial for fabricating new types of biomaterials (Ramberg et al. 2021b).

This review summarizes methods for controlling protein assembly and crystallization, with a focus on synthetic, water-soluble macrocycles that function as *molecular glues*. Such macrocycles are versatile, enabling the assembly of diverse protein targets without the requirement for natural ligand-binding sites or pervasive protein engineering. As a foundation to the review, we summarize general crystal engineering techniques and the key terminology.

## Crystal engineering

*Polymorphs* are different crystal forms of the same components, and* polymorph searching* can provide diverse materials from few building blocks. Polymorphs are important because different packing arrangements of the same molecules can have distinct physical properties with significant implications, for example, in pharmaceutical bioavailability (Griesser 2006; Desiraju 2008). While polymorphs are defined as chemically identical, difficulties arise with multi-component systems. Pseudopolymorphism describes crystals with slightly different chemical compositions or stoichiometries (Desiraju 2004). The strict definition of polymorphism does not hold for proteins since the total solvent and ion content vary in different crystal forms (Ulrich and Pietzsch 2015). Additionally, host–guest cocrystals (e.g. protein–macrocycle polymorphs) may vary in the ratio of their components (Rennie et al. 2018). Nonetheless, polymorphism is an accepted term for describing different crystalline arrangements of a protein (Van Driessche et al. 2018; Lanza et al. 2019). Regardless of definitions, polymorphs, pseudopolymorphs, salts and solvates are different points on a structural landscape (Blagden and Davey 2003; Desiraju 2008).

Desiraju defined crystal engineering by the exchange of *supramolecular synthons*, reproducible structural units mediated by intermolecular interactions, between one structure and another (Desiraju 1995; Nangia and Desiraju 2019). The larger the sample of crystal structures, the easier it is to recognize patterns and identify synthons. Series of related crystalline frameworks can be generated when a molecular component (tecton) is replaced by an analogue, provided that the supramolecular synthons are conserved. *Tecton* was introduced by Wuest in 1991, describing building blocks that predictably self-assemble or co-assemble with other molecules, and *molecular tectonics* is ‘the art and science of supramolecular construction using tectonic subunits’ (Simard et al. 1991; Wang et al. 1994; Hosseini 2005). The predictable/reproducible assembly points of tectons are supramolecular synthons, formed by one or more intermolecular interactions (e.g. H-bonds, cation–*π* interactions, etc.) (Hosseini 2005). In this review, we explore the identification and application of supramolecular synthons in protein frameworks.

## Modular frameworks

A fundamental principle of (crystal) engineering is modularity—the ability to adjust or mix-and-match building blocks in a predictable fashion. This approach is instrumental in porous molecular frameworks, with metal–organic frameworks (MOFs) and covalent organic frameworks (COFs) at the forefront due to their strong, coordination or covalent bonding, respectively. MOF engineering is particularly well established (Furukawa et al. 2013; Feng et al. 2019), with numerous examples of accurate framework design. MOFs are predictably assembled by organic linkers coordinating metal ions or clusters (nodes) with a defined coordination geometry. Yaghi and coworkers reported a series of isoreticular MOFs based on MOF-5 (Li et al. 1999; Eddaoudi et al. 2002). By substituting the organic linker with structurally similar units, framework porosity was tuned while topology was maintained. This widely adopted modular approach allows for the predictable construction of MOF series with various properties and applications (Yuan et al. 2010; Deng et al. 2012). Likewise, isoreticular series of COFs are generated by substituting structurally similar modules (Biswal et al. 2013; Geng et al. 2020). A ‘mix-and-match’ assembly can also generate crystalline frameworks using porous organic cages (POCs). Cooper and coworkers demonstrated the modular assembly of organic cages into computationally predictable porous cocrystals (Jones et al. 2011; Liu et al. 2019).

**Fig. 1:**
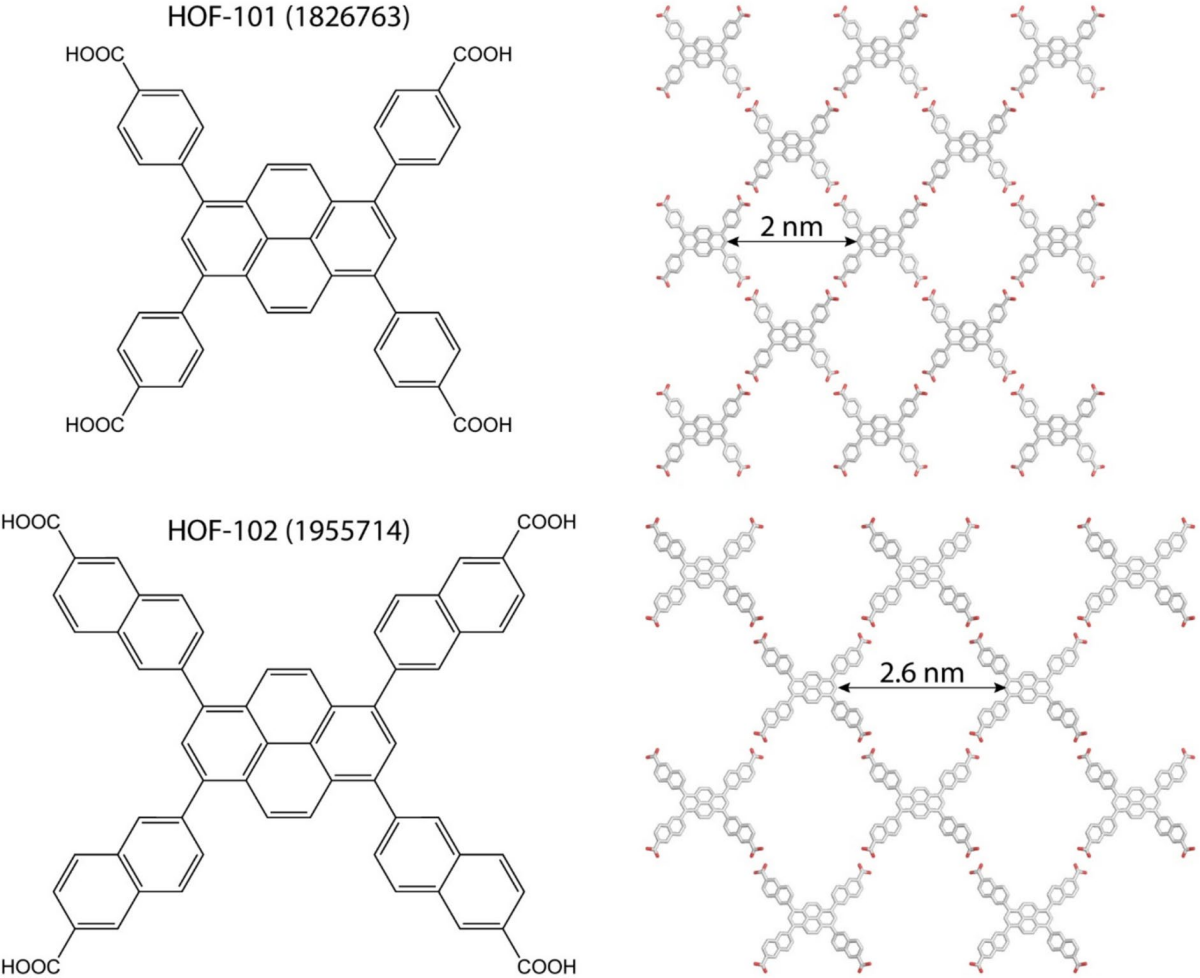
Symmetric tectons direct isostructural frameworks HOF-101 and HOF-102 with different pore diameters. The frameworks are mediated by inter-tecton *π − π* stacking (not shown) and hydrogen bonding. CCDC numbers are indicated

Hydrogen-bonded organic frameworks (HOFs) may be most relevant to protein crystals. Compared to the directional coordinate or covalent bonds of MOFs and COFs, respectively, the hydrogen bonds that govern HOF assembly are weak. Such weak intermolecular bonds, as typical also of protein crystals, reduce framework robustness and predictability (Little et al. 2020; Li et al. 2020). Early work by Wuest was fundamental for identifying suitable tectons that direct predictable ‘open’ frameworks (Simard et al. 1991; Wang et al. 1994; Brunet et al. 1997; Maly et al. 2007). Wuest proposed that a suitable tecton comprises a rigid core that arranges multiple peripheral (‘sticky’) groups in a defined geometry. These sticky groups engage in directional inter-tecton hydrogen bonding according to established motifs (i.e. supramolecular synthons). For example, rigid tetrahedral tectons with four peripheral sticky groups formed open, diamondoid networks (Simard et al. 1991; Wang et al. 1994). If a tecton is designed appropriately, the formation of multiple directional hydrogen bonds may be favoured over tight crystal packing, yielding porous frameworks. The early 2010 s marked a turning point in HOF design and functionality, with the first examples of permanently porous HOFs (Brunet et al. 1997; Yang et al. 2010; He et al. 2011). Combining hydrogen bonding with additional noncovalent inter-tecton interactions, such as *π − π* stacking, afforded frameworks with increased rigidity (e.g. Fig. 1) as well as enabling modular construction of isostructural HOFs (Hashim et al. 2018; Yin et al. 2018; Wang et al. 2020; Suzuki et al. 2021). Tectons can be altered/substituted, while maintaining the supramolecular synthons that dictate assembly. For example, isostructural HOF-101 (Yin et al. 2018) and HOF-102 (Wang et al. 2020) were generated using *C*_4_-symmetric pyrene-1,3,6,8-tetraaryl-based tectons with different substituent lengths, tuning the framework porosity (Fig. 1).

## Protein engineering for crystal design

Controlled protein assembly is challenging due to the chemical and topological heterogeneity of protein surfaces. Nevertheless, several protein crystal engineering methods have been developed (Zhu et al. 2021; Kojima et al. 2022). In this section, we discuss how protein engineering is employed to (1) create protein tectons that form novel (porous) frameworks, or, (2) modify and functionalize known/existing protein crystals.

**Fig. 2:**
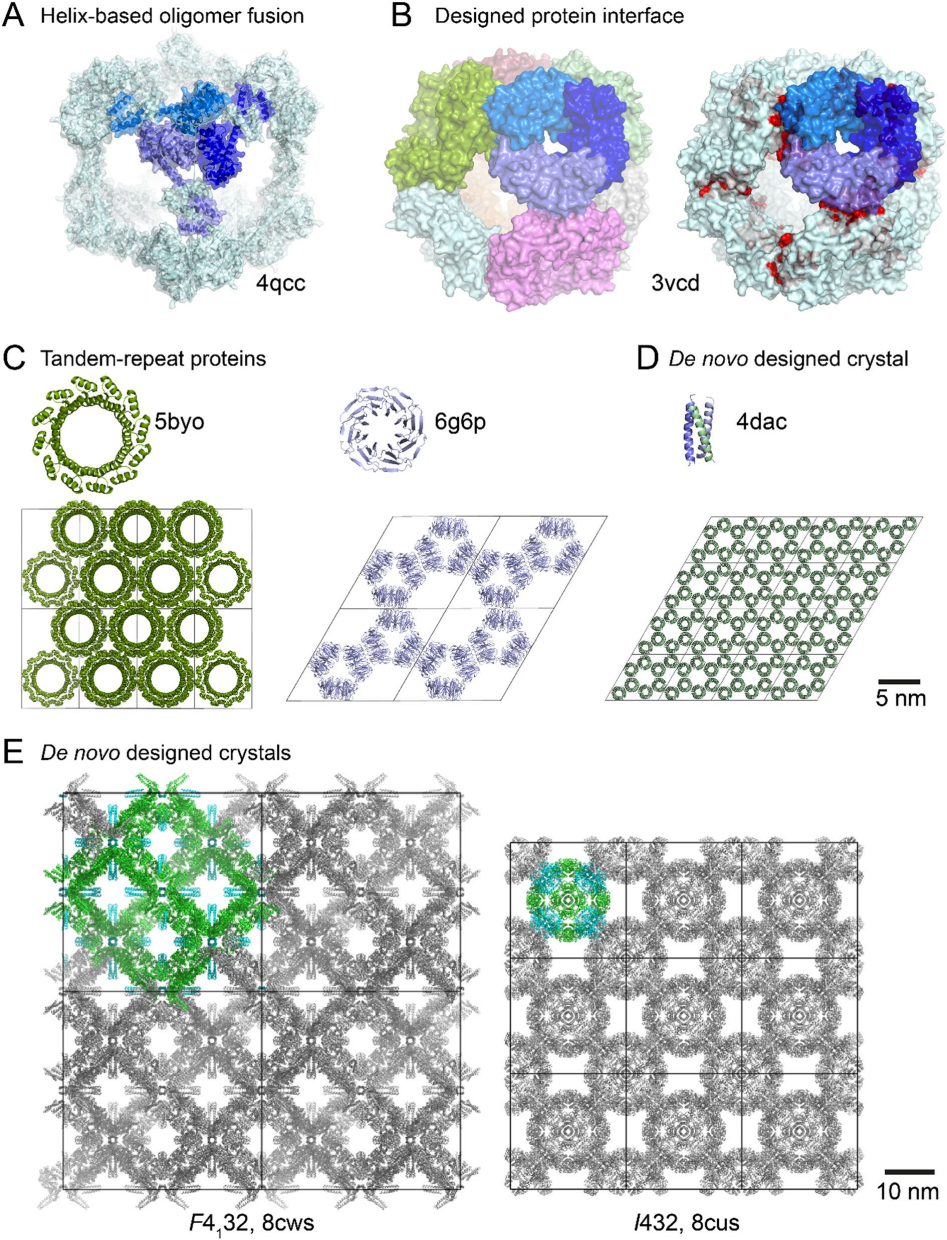
Protein engineering. **A** A cubic cage of α-helix fused trimeric and dimeric proteins. **B** An octahedral cage formed by self-assembled trimers. Mutated residues at the designed protein–protein interface are red. **C** De novo designed tandem-repeat protein tectons. **D** The first reported de novo designed protein crystal. **E** De novo designed porous protein crystals comprising tetrahedral or octahedral cages (coloured). PDB codes are indicated

Parallel to the molecular tectonics strategies used for generating porous HOFs, symmetric proteins can serve as tectons for controlled assembly (Yeates et al. 2016). A high degree of symmetry means that fewer inter-tecton interfaces are required to direct assembly. Yeates and coworkers used natural protein oligomers, homodimers and homotrimers, as building blocks to design and construct frameworks. With their ‘helix-based oligomer-fusion strategy’, a dimeric domain and a trimeric domain were joined by a rigid α-helix, forming a self-assembling fusion protein (Padilla et al. 2001; Lai et al. 2014). The protein domains are linked in a specific orientation, resulting in a designed architecture upon oligomerization. For example, a cubic cage comprising 24 fusion proteins (PDB 4qcc, Fig. 2A) comprises trimers at the vertices and dimers along the edges (Lai et al. 2014).

D*e novo* protein design has accelerated in recent years, with the development of software such as Rosetta (Huang et al. 2016). Baker and coworkers have accurately designed finite tetrahedral, octahedral or icosahedral protein cages using natural homo-oligomers that share symmetry elements with the target assembly. For example, a designed octahedral cage was made from eight homotrimers providing the threefold rotational symmetry at the vertices of a cube (King et al. 2012). A single Rosetta-designed protein–protein interface, nine mutations away from the natural protein scaffold (PDB 3n79), was sufficient to promote assembly of the trimers into the desired cage. Cage fabrication was confirmed by size exclusion chromatography (SEC) and X-ray crystallography (PDB 3vcd, Fig. 2B). A similar strategy yielded a tetrahedral cage, and later, two-component tetrahedral or icosahedral cages (King et al. 2014; Bale et al. 2016). For example, a 120-subunit icosahedral cage comprised 12 homopentamers and 20 homotrimers aligned along the fivefold and threefold icosahedral symmetry axes, respectively (Bale et al. 2016). In general, the designed interfaces resemble those found in nature, comprising hydrophobic cores flanked by polar residues.

De novo designed symmetric monomeric proteins have also yielded porous crystals (Doyle et al. 2015; Noguchi et al. 2019). Bradley and coworkers designed tandem repeat proteins with closed toroidal structures (Doyle et al. 2015). The inner pore of the protein was tuneable by varying the number of repeat units: a 9-repeat monomer had a 2.5 nm diameter pore, while a 12-repeat monomer had a 3 nm pore (PDB 4yxz and 5byo, respectively). These pores formed the solvent channels in the crystals due to toroid stacking (Fig. 2C), so crystal porosity was tuneable by protein design. Voet and coworkers designed tandem-repeat β-propellers by reverse-engineering natural proteins. Monomeric designs included a *C*_6_-symmetric 6-bladed β-propeller, *Pizza6* (PDB 3ww9) (Voet et al. 2014), and *C*_8_-symmetric 8-bladed β-propellers, *Tako8* and *Ika8* (PDB 6 g6 m and 6 g6p) (Noguchi et al. 2019). The designed proteins self-assembled to yield crystalline frameworks, with the *Ika8* protein yielding a particularly porous assembly with 66% solvent content and 9 nm pore diameters (Fig. 2C).

Despite progress in protein design, the accurate de novo design of 3D protein crystals with predetermined space groups remains challenging. Saven and coworkers engineered the first de novo protein crystal from a ~ 3 kDa designed coiled-coil homotrimer (Lanci et al. 2012). By introducing lateral and vertical protein–protein contacts, the trimeric tectons predictably assembled into a framework in space group *P*6 (PDB 4 dac, Fig. 2D). Recently, the Baker lab developed a hierarchical design strategy for fabricating de novo 3D protein crystals (Li et al. 2023). Pairs of designed protein oligomers first form tetrahedral or octahedral cages, which then assemble into the predicted crystal packing (space groups *F*4_1_32 or *I*432 in PDB 8cws or 8cus, respectively, Fig. 2E). The assembly of the designed oligomers occurred first, followed by cage formation and finally inter-cage packing. This stepwise assembly was controlled by fine-tuning the interaction strength of each step, with the strongest interactions occurring first followed by the weaker interactions. The engineered crystalline frameworks were highly porous with up to 90% solvent content, and stable. Moreover, the design was modular as the unit cell parameters (and crystal porosity) could be tuned by varying the length of a protein component, as confirmed by small angle X-ray scattering (SAXS).

Protein engineering can also be applied to modify natural protein crystals, altering or enhancing their functionality (Koshiyama et al. 2010; Abe et al. 2015, 2017; Huber et al. 2018; Maita 2018; Nguyen et al. 2019, 2021; Ernst et al. 2019; Heater et al. 2020; Kojima et al. 2022, 2023). Particular progress has been achieved with the cypovirus protein polyhedra PhC (Coulibaly et al. 2007) as recombinant PhC can encapsulate foreign proteins by co-expressing polyhedrin and the target protein in insect cells. For immobilization in the PhC crystal, the target protein is N-terminally tagged with the H1-helix of polyhedrin that enables interaction with the crystal protomers. Applying this strategy, Ueno and coworkers designed a polyhedrin variant capable of long-term enzyme storage (Abe et al. 2015). The crystal was engineered to dissolve at a suitable pH thus, releasing the active enzyme protein kinase C. Wild-type PhCs dissolve at pH ≥ 10.5, where enzymatic activity is typically lost, so dissolution at a lower pH required a reduction in crystal stability. Arg13 was identified as a key contributor to crystal stability as it donates four intermolecular hydrogen bonds to a protein–protein interface. The R13A mutation removing the hydrogen bond donor destabilized this interface, resulting in crystal dissolution at pH 8.5.

Alternatively, PhC can function as a solid biocatalyst by immobilizing enzymes. Crucially, it is possible to make deletion mutants of polyhedrin that crystallize in the same lattice as the wild-type protein (space group *I*23), but with extended pores (Abe et al. 2017; Nguyen et al. 2021). These porous variants can incorporate exogenous proteins in vivo. Ueno and coworkers have reported a 38-residue deletion mutant of polyhedrin that forms a crystal with pores of 5 nm diameter (PDB 6 lee). Two H1-tagged enzymes *Candida antarctica* lipase B (CALB) and *Lactobacillus kefir* alcohol dehydrogenase (ADH) were immobilized in the crystal, forming a catalytic vessel for a cascade reaction. CALB catalyzed the hydrolysis of the substrate 7-(3,4-diacetoxybutyloxy)−4-methyl-2*H*-chromen-2-one to form an intermediate. The dehydrogenation reaction of the intermediate was catalyzed by ADH, followed by an elimination reaction at pH > 6.5 to give the blue fluorescent product 4-methylumbelliferone. The reaction substrate and intermediate could diffuse efficiently through the extended pores of the crystal, resulting in an almost fourfold increased reactivity relative to mixtures of the free enzymes (Nguyen et al. 2021).

Protein engineering facilitates the development of protein frameworks through the precise design of self-assembling tectons. Alternatively, mutagenesis can be employed to modify the properties of natural protein crystals (e.g. stability or porosity), thereby modulating their functionality (e.g. enzyme storage, solid-state catalysis). To reduce reliance on potentially labour-intensive protein engineering, an alternative strategy is to incorporate an additional component, such as a metal ion or ligand, to direct protein assembly.

## Protein-based MOFs

Metal coordination sites, especially zinc binding, can direct protein assembly as is abundantly evident in the Protein Data Bank. Metal binding sites enable predictable assemblies via directional coordinate bonds, which are stronger than the noncovalent interactions that govern typical protein–protein interfaces. In an early example, Tezcan and coworkers engineered the monomeric cytochrome *cb*_*562*_ to assemble into functional oligomers or crystals via controlled metal coordination (Ni and Tezcan 2010; Brodin et al. 2012; Song and Tezcan 2014; Golub et al. 2020).

**Fig. 3:**
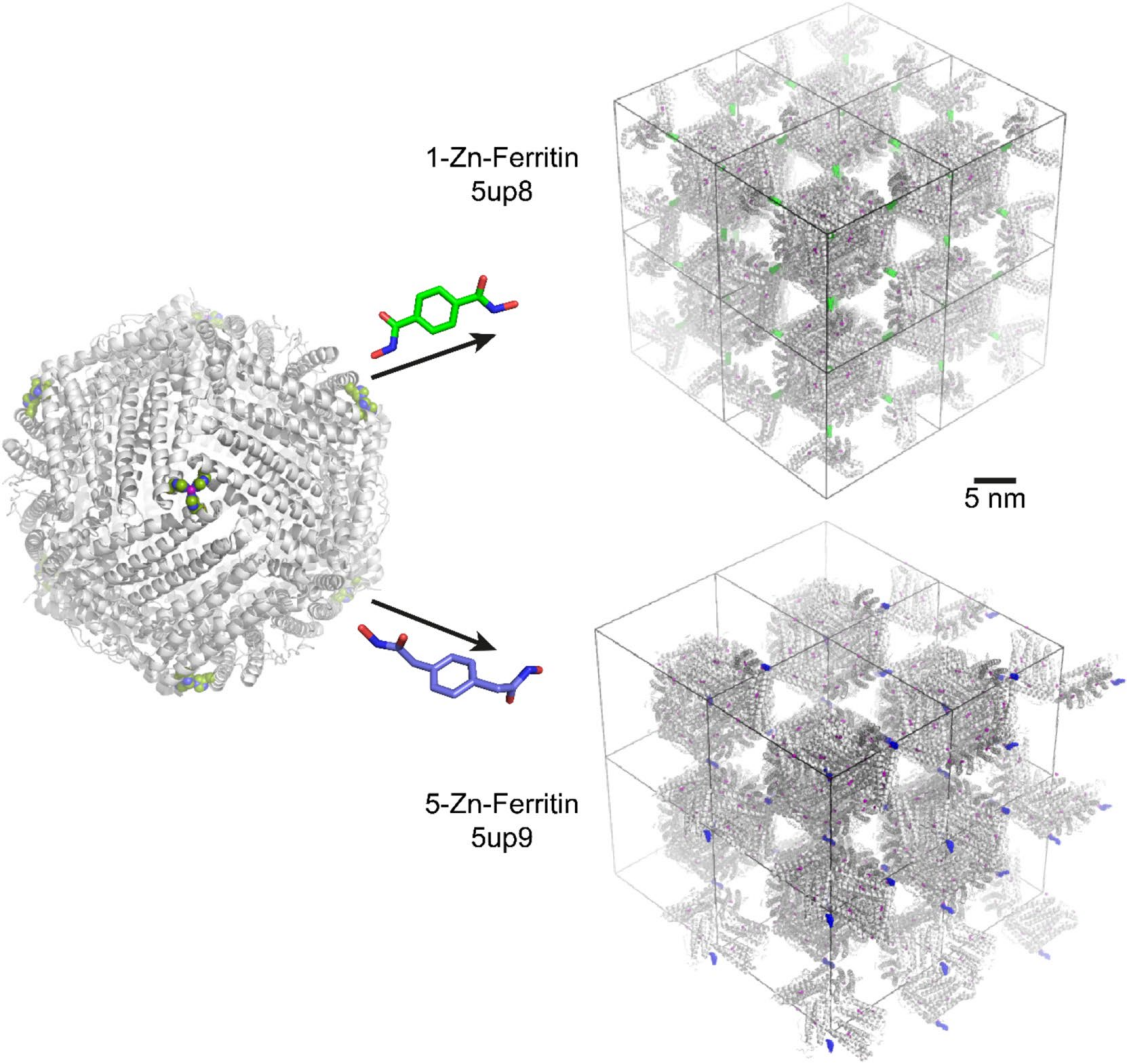
Modular ferritin MOFs. The T122H mutation (green spheres) enables Zn binding (magenta), with eight binding sites per quasi-spherical protein node. Ferritin assembly is mediated by ditopic ligands, for example benzene-1,4-dihydroxamic acid (green) or xylene-1,4-dihydroxamic acid (blue), yielding frameworks 1 − Zn − ferritin (space group *I*432, PDB 5up8) and 5 − Zn − ferritin (space group *I*4, PDB 5up9), respectively

The strategic insertion of metal binding sites on protein surfaces coupled with coordination by organic linkers can result in protein-based MOFs (Sontz et al. 2015; Bailey et al. 2017; Bailey and Tezcan 2020). A case in point involves the quasi-spherical, octahedral 24-mer human heavy-chain ferritin functioning as a tecton. Eight metal (Zn^2+^) binding sites, in a cubic arrangement, were engineered at the surface exposed *C*_3_-symmetric pores of the protein via the T122H mutation (Fig. 3). Each engineered site enabled tetrahedral Zn coordination, with the base comprising three His122 residues and a single exposed coordination site. These engineered metal binding sites served as nodes, while ditopic organic ligands with hydroxamic acid functional groups served as linkers, coordinating the Zn and directing the designed body-centred cubic lattice of ferritin. Remarkably, the modularity of conventional MOF design is also applicable in generating ferritin MOFs. By varying the metal or ditopic organic linker, a library of frameworks with predictable structures and tuneable properties was synthesized (Fig. 3).

## Protein assembly/crystallization via native ligand interactions

Protein assembly can also be controlled by ligand binding (Fegan et al. 2010; Zhu et al. 2021). Here, proteins with specific ligand-binding sites serve as the tectons for assembly and/or crystallization, involving minimal or no protein engineering. Hayashi and coworkers demonstrated the programmed assembly of hemeproteins via non-native heme–protein interactions (Kitagishi et al. 2007; Oohora et al. 2012). Supramolecular homopolymers of cytochrome *b*_562_ were generated using a self-complementary apo-protein modified with an ‘external’ heme (Kitagishi et al. 2007). The H63C mutation on the protein surface opposite the heme pocket enabled covalent attachment of a modified heme. The intrinsic heme was removed from the heme pocket by acid-denaturation of the protein. Linear polymerization of the modified apo-protein was confirmed by SEC and atomic force microscopy (AFM). We reported a different approach to supramolecular polymers with cytochrome *c* and two different calixarenes binding at distinct surface patches, each mediating *C*_2_-symmetric dimers of the protein (Mockler et al. 2021a). Here, modification of the protein or specific ligand-binding pockets was not required, suggesting that this technique may be applicable with a range of protein targets.

**Fig. 4:**
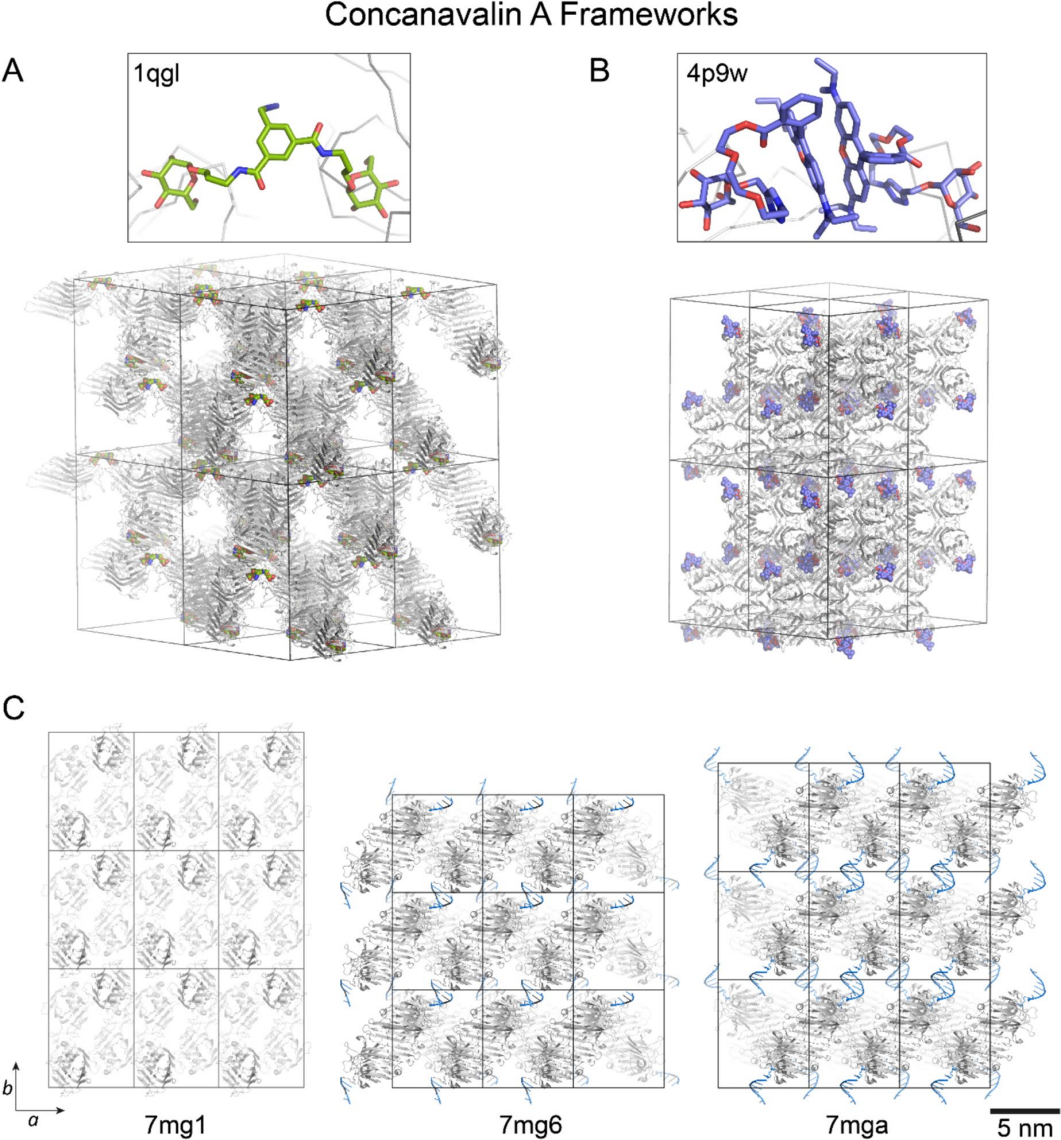
ConA crystals mediated by **A** a ditopic saccharide linker or **B** Rh3Man. **C** Crystals of ConA in the absence (PDB 7 mg1) or presence (PDB 7 mg6, 7 mga) of DNA glycoconjugates. Increasing the length of the DNA sequence results in increased spacing between protein sheets, i.e. expansion along the* b* axis

Concanavalin A (ConA) is a homotetramer, with four mannose binding sites located at the vertices of a tetrahedron. Various laboratories have manipulated this lectin–sugar interaction to direct protein frameworks and engineer protein crystals. Freeman and coworkers generated a ‘diamondlike’ assembly of ConA using a ditopic saccharide linker (Dotan et al. 1999). They predicted that assembly of the near-tetrahedral protein by a *C*_2_-symmetric ligand (bismannopyranoside) would yield a cubic crystal with diamondlike packing. This assembly was confirmed by electron microscopy and X-ray crystallography at low resolution. The crystallographic data were sufficient to confirm the ‘pseudo-cubic’ unit cell (*a* = 200, *b* = 204,* c* = 208 Å), similar to the predicted lattice (*a* = *b* = *c* = 202 Å) (Dotan et al. 1999). Naismith, Toone and coworkers reported a porous cocrystal of ConA with a different ditopic saccharide linker (PDB 1qgl, Fig. 4A) in which tetramer assembly was mediated primarily by the designed linker (Dimick et al. 1999). Later, Chen, Jiang and coworkers employed dual supramolecular interactions to direct ConA assembly (Sakai et al. 2014; Hu et al. 2018). A combination of lectin–sugar binding and rhodamine dimerization directed framework fabrication (Fig. 4B). The sugar (α-d-mannopyranoside or α-d-glucopyranoside) and rhodamine were connected by a flexible oligo(ethylene glycol) spacer, yielding bivalent ligands (e.g. Rh3Man) that mediated ConA frameworks, with polymorph selection apparently depending on the crystallization method.

Mirkin and coworkers have employed DNA self-assembly (hybridization) to direct protein assembly and crystallization (Winegar et al. 2020; Partridge et al. 2021). In one example, DNA oligonucleotides conjugated with mannose were used to generate modular ConA crystalline frameworks (Partridge et al. 2021). In the absence of the DNA glycoconjugates, ConA crystallized in the known space group *I*222 (PDB 7 mg1, Fig. 4C) in which the sugar binding site contributes to protein–protein interactions. Binding of the bulky mannose-DNA conjugate sterically blocked the residues around the sugar-binding site, disrupting the native assembly. Cocrystallization of ConA with complementary (e.g. Man-TTTT and Man-AAAA) or self-complementary (e.g. Man-AGCT) mannose-DNA conjugates yielded crystals in space group *P*2_1_22 (PDB 7 mg9 and 7 mg6) with the self-assembling glycoconjugates directing the crystal packing. Only (self-) complementary DNA sequences were effective. Non-complementary sequences failed to yield crystals. Thus, dual supramolecular interactions, DNA hybridization and ConA–mannose binding, mediated the assembly. Mirkin and coworkers also demonstrated system modularity, in which different DNA sequences could be incorporated to programme the crystal structure. Increasing the DNA length from four to six base pairs yielded crystals in the same space group but with a ∼7 Å expansion of the unit cell along the *b-*axis (PDB 7 mga, Fig. 4C). In each structure, the proteins pack in identical sheets, with the distance between the sheets dependent on the DNA length.

These strategies with lectins afford protein crystal engineering without the need for mutagenesis. However, the methods require the synthesis of functionalized glyco-ligands and are limited to lectins. An alternative approach is to use commercially available synthetic macrocycles applicable to various protein targets.

## Protein–macrocycle recognition and assembly

Predictable protein surface recognition by macrocycles provides a means for controlling protein assembly, circumventing the need for designed protein–protein interfaces or natural ligand/metal binding sites. The protein recognition properties of water-soluble cucurbiturils (Armstrong et al. 2024), calixarenes (Crowley 2022), crown ethers (Lee et al. 2014) and porphyrins (Goel et al. 2005) make them versatile receptors for a wide range of protein targets.

Macrocycles act as molecular glues by simultaneously binding two or more protein surfaces, thereby directing their co-assembly. These glues can function independently or in conjunction with other methods, including protein–metal coordination (Flood et al. 2022; Guagnini et al. 2020a) or protein engineering (Guagnini et al. 2020b; Mockler et al. 2025a, 2025b; Ramberg et al. 2021a). In the latter case, point mutations or minimal affinity tags (1–3 residues) may be sufficient to ensure controlled, site-specific binding, without impacting protein structure/function (Armstrong et al. 2024). The following section focuses on protein recognition and assembly by cucurbiturils and calixarenes. In the subsequent sections, we highlight reproducible protein–macrocycle and macrocycle–macrocycle interfaces (supramolecular synthons) that show promise in protein crystal engineering.

## Predictable protein recognition: protein–macrocycle synthons

**Fig. 5:**
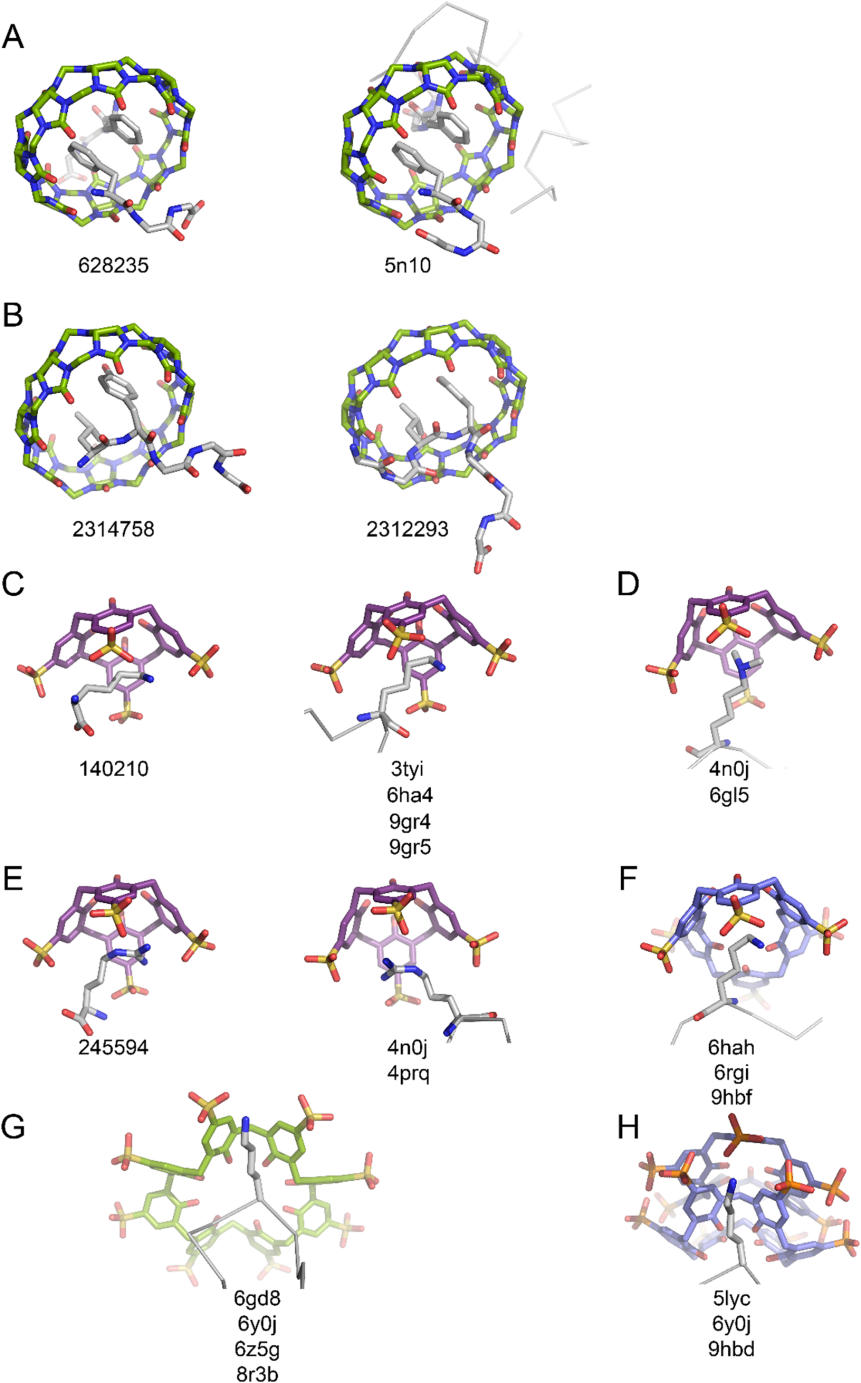
Reproducible structural units (supramolecular synthons) in crystal structures with amino acid/peptide/protein-binding macrocycles. CCDC and PDB codes are indicated. **A** Phe-Gly-Gly–**Q8**, **B** Leu-Tyr–**Q8**, **C** lysine–**sclx**_**4**_, **D** dimethyllysine–**sclx**_**4**_, **E** arginine–**sclx**_**4**_, **F** lysine–**sclx**_**6**_ or **pclx**_**6**_, **G** lysine–**sclx**_**8**_, **H** lysine–**pclx**_**6**_ dimer. Carbons are coloured: green (**Q8** and **sclx**_**8**_), purple (**sclx**_**4**_) or mauve (**sclx**_**6**_ or **pclx**_**6**_). See Table 1 for details

Here, we introduce the two most important synthetic macrocycles currently used for protein recognition, describing the key synthons (Fig. 5, Table 1).

**Table 1:** Supramolecular synthons with protein-binding macrocycles

Supramolecular synthon	Macrocycle conformation	Representative structures	PDB/CCDC
Lysine–**sclx**_**4**_^a^	Bowl	l-lysine–**sclx**_**4**_	140210
		Cytochrome* c*–**sclx**_**4**_	3tyi
		PAF–**sclx**_**4**_	6ha4
		MKA-RSL–**sclx**_**4**_	9gr4
		MKAA-RSL–**sclx**_**4**_	9gr5
Lysine–**sclx**_**6**_ or **pclx**_**6**_^a^	1,2,3-alternate double cone	PAF–**sclx**_**6**_	6hah
		Cytochrome *c*–**sclx**_**6**_	6rgi
		MK-RSL–**pclx**_**6**_	9hbf
Lysine–**pclx**_**6**_** dimer**^a^	Double cone	Cytochrome *c*–**pclx**_**6**_	5lyc
		Cytochrome *c*–**pclx**_**6**_–**sclx**_**8**_	6y0j
		RSL–**pclx**_**6**_	9hbd
Lysine–**sclx**_**8**_^a^	Pleated loop	Cytochrome* c*–**sclx**_**8**_ *P*3_1_	6gd8
		Cytochrome* c*–**sclx**_**8**_ *P*4_3_2_1_2	6gd9
		Cytochrome *c*–**pclx**_**6**_–**sclx**_**8**_	6y0j
		RSL–**sclx**_**8**_ *I*23	6z5g
		RSL-D32N–**sclx**_**8**_ *I*23	8q6b
		Pent–**sclx**_**8**_ *P*4_3_2_1_2	8r3b
		Pent–**sclx**_**8**_ *P*2_1_	8r3c
Dimethyllysine–**sclx**_**4**_^a^	Bowl	HEWL*–**sclx**_**4**_	4n0j
		RSL*–**sclx**_**4**_	6gl5
Arginine–**sclx**_**4**_^a^	Bowl	d-arginine–**sclx**_**4**_	245594
		HEWL–**sclx**_**4**_	4prq
		HEWL*–**sclx**_**4**_	4n0j
**pclx**_**6**_–**pclx**_**6**_^b^	Double cone	Cytochrome *c*–**pclx**_**6**_	5lyc
		Cytochrome *c*–**pclx**_**6**_–**sclx**_**8**_	6y0j
		RSL–**pclx**_**6**_	9hbd
**sclx**_**8**_–**sclx**_**8**_^b^	Pleated loop	Na-**sclx**_**8**_ salt	2298745
		RSL–**sclx**_**8**_ *I*23	6z5g
		RSL-D32N–**sclx**_**8**_ *I*23	8q6b
Dimethyllysine–**Q7**^a,c^	Doughnut	RSL*–**Q7** *C*222_1_	6f7w
		RSL*–**Q7** *F*432	6f7x
		RSLex*–**Q7**	6su0
Phe-Gly-Gly–**Q8**^a^	Doughnut	2:1 FGG–**Q8**	628235
		14-3-3 adapter protein–FGG-ERα–**Q8**	5n10
Leu-Tyr–**Q8**^a^	Doughnut	GGLYGGG	2312293
		LYGGG	2314758

^a^See Fig. 5

^b^See Fig. 10

^c^Dimethyllysine can adopt alternate conformations in the **Q7** cavity

*Indicates chemically dimethylated protein

Cucurbit[n]urils (**Qn**, where* n* = 6, 7, 8) are donut-shaped macrocycles comprising *n* glycoluril units linked by bis(methylene) bridges (Fig. 5). **Qn** hosts can accommodate different guests with high affinity, depending on the cavity size and the nature of the guest (Lagona et al. 2005; Barrow et al. 2015; Armstrong et al. 2024). Freeman, Mock and Shih first reported **Q6** and its capacity to bind organic amines, with the alkyl or aryl portion inside the **Q6** cavity and the ammonium ion(s) forming ion–dipole bonds with the carbonyl rim (Freeman et al. 1981; Mock and Shih 1983, 1986; Freeman 1984). **Q7** can accommodate larger guests than **Q6**, and **Q8** can accommodate even larger guests or two guests simultaneously (Kim et al. 2000, 2001; Lee et al. 2003). In general, the high affinity inclusion of nonpolar guests within the **Qn** cavity is enabled by the displacement of high-energy water molecules (Biedermann et al. 2014), while cationic groups interact with the carbonyl rims. This combination of binding features makes **Qn** suitable hosts for peptides or proteins, in particular, N-terminal sites (Armstrong et al. 2024).

As observed with small molecule guests, amino acid residue encapsulation depends on the **Qn** size enabling selective binding (Barrow et al. 2015; Armstrong et al. 2024) and suggests the existence of residue–**Qn** synthons (Fig. 5). Several examples of site-specific peptide or protein binding by **Q6**, **Q7** or **Q8** have been demonstrated. Importantly, the recognition motifs discovered with short peptides are transferable to folded proteins. For example, Urbach and coworkers demonstrated the recognition of Met-terminated peptides by **Q8** (Hirani et al. 2018). Isothermal titration calorimetry (ITC) studies revealed high affinity binding of **Q8** to tripeptides MFA, MYA, MLA or MKA (*K*_d_ ∼0.14–2.6 µM). In some cases, peptide folding enabled the simultaneous inclusion of the N-terminal methionine and the adjacent residue in the **Q8** cavity, a binding mode known as the *pair-inclusion* motif (Hirani et al. 2018; Suating et al. 2024a, 2024b). In contrast to **Q8**, a single residue can be encapsulated by **Q6**. We reported **Q6** recognition of the N-terminal Met-Lys motif in the model protein SAMP2 (Ramberg et al. 2021a). Transfer of this Met-Lys binding tag to RSL (the MK-RSL mutant) also resulted in site-specific interaction. Selective and high affinity binding at the N-terminus was evidenced by NMR experiments. Recently, this N-terminal Met-Lys macrocycle binding tag has been developed further using *p*-sulfonato-calix[4]arene (**sclx**_**4**_) and *p*-phosphonato-calix[6]arene (**pclx**_**6**_) (Mockler et al 2025a, 2025b).

The **Q7** cavity can encapsulate bulky residues, including methylated lysines or arginines (Gamal-Eldin and MacArtney 2012; Guagnini et al. 2018, 2020a, b; Ramberg et al. 2021c; Ramberg and Crowley 2023) or N-terminal aromatics (Rekharsky et al. 2008; Chinai et al. 2011; Armstrong et al. 2024). **Q7** is more water-soluble than **Q6** and **Q8**, making it more useful for protein binding. In 2011, Urbach and coworkers reported a cocrystal structure of human insulin and **Q7** (PDB 3q6e) (Chinai et al. 2011). The asymmetric unit comprised two insulin molecules, one of which was bound by the macrocycle. As predicted from studies with short peptides (Rekharsky et al. 2008), **Q7** bound the N-terminal phenylalanine of the insulin B-chain. In the crystal structure, the phenylalanine side chain is buried in the **Q7** cavity while the N-terminal ammonium forms an ion–dipole interaction with the carbonyl portal. This binding mode is specific to the N-terminal phenylalanine (Chinai et al. 2011).

**Q8** is also a receptor for N-terminal aromatics (Bush et al. 2005; Heitmann et al. 2006; Nguyen et al. 2010; Hou et al. 2013; Bosmans et al. 2016; de Vink et al. 2017; Armstrong et al. 2024) In 2006, Urbach and coworkers reported a ternary cocrystal structure of **Q8** and two Phe-Gly-Gly tripeptides (Heitmann et al. 2006). The **Q8** cavity simultaneously accommodated the two Phe side chains, while each N-terminal ammonium formed ion–dipole bonds with a carbonyl portal (CCDC 628235, Fig. 5A). Utilizing this binding tag, **Q8** can dimerize (or polymerize) proteins (Nguyen et al. 2010; Hou et al. 2013; Bosmans et al. 2016; de Vink et al. 2017). Brunsveld and coworkers demonstrated the **Q8**-mediated assembly of proteins that were N-terminally tagged with the Phe-Gly-Gly motif (Nguyen et al. 2010). Protein dimerization was reversible by the addition of the competing ligand methyl viologen. In a crystal structure of the 14-3-3 adapter protein with Phe-Gly-Gly-ERα and **Q8** (PDB 5n10, Fig. 5A), the macrocycle bound two Phe-Gly-Gly motifs as previously described by Urbach and coworkers (de Vink et al. 2017). This Phe-Gly-Gly–**Q8** supramolecular synthon is an effective affinity tag with potential applications in protein crystal engineering. Recently, Urbach and coworkers provided crystallographic evidence of **Q8** encapsulating N-terminal or non-terminal Leu-Tyr motifs (CCDC 2314758 and 2312293, Fig. 5B) (Suating et al. 2024a, b). Although differences arise due to the presence or absence of ion–dipole interactions, **Q8** encapsulation of the Leu-Tyr pair is similar in the distinct structures and is a supramolecular synthon.

Calix[*n*]arenes are *n* para-substituted phenol units linked by methylene bridges. In 1979, the first crystallographic evidence of calixarenes also provided the initial demonstration of their host–guest capabilities (Andreetti et al. 1979). The cocrystal structure of *tert*-butyl-calix[4]arene encapsulating toluene was described as a *clathrate*, highlighting the ability of calixarenes to trap guests. Shinkai’s development of the anionic and water-soluble *p*-sulfonato-calix[n]arenes (**sclx**_**n**_) (Shinkai et al. 1986), and later Raston’s *p*-phosphonato-calix[n]arenes (**pclx**_**n**_) (Clark et al. 2008; Dziemidowicz et al. 2008; Martin and Raston 2011) facilitated interactions with amino acids, peptides or proteins in water, similar to anionic molecular tweezers (Fokkens et al. 2005; Sinha et al. 2011). **sclx**_**4**_, locked in the bowl conformation, can *encapsulate* one amino acid side chain. In the early 2000 s, cocrystal structures of **sclx**_**4**_ encapsulating lysine (CCDC 140210), alanine (CCDC 154340), histidine (CCDC 167829), phenylalanine (CCDC 167831), tyrosine (CCDC 167832) or arginine (CCDC 245594) were reported (Selkti et al. 2000; Atwood et al. 2002; Lazar et al. 2004). In the **sclx**_**4**_–lysine cocrystal structure, the lysine C^ε^ forms cation–*π* bonds with three phenol rings while the N^ζ^ ammonium salt bridges two sulfonates. In 2012, we reported the first protein–calixarene cocrystal structure: the cationic yeast cytochrome *c* (p*I* ∼9.5) in complex with **sclx**_**4**_ (McGovern et al. 2012). Three lysine side chains on the protein surface were encapsulated by calixarenes, binding as previously described by Selkti et al. (PDB 3 tyi, Fig. 5C). This lysine–**sclx**_**4**_ synthon reoccurs in multiple protein–calixarene cocrystal structures (McGovern et al. 2012; Doolan et al. 2018; Alex et al. 2019, 2020; Mockler et al. 2025a). **sclx**_**4**_ can also bind dimethyllysine (McGovern et al. 2014b) or arginine (McGovern et al. 2014a, 2014b) (Fig. 5D, E). Most recently, we have provided crystallographic evidence of **sclx**_**4**_ encapsulating the N-terminal methionine in the protein MK-RSL (Mockler et al. 2025a).

**Fig. 6:**
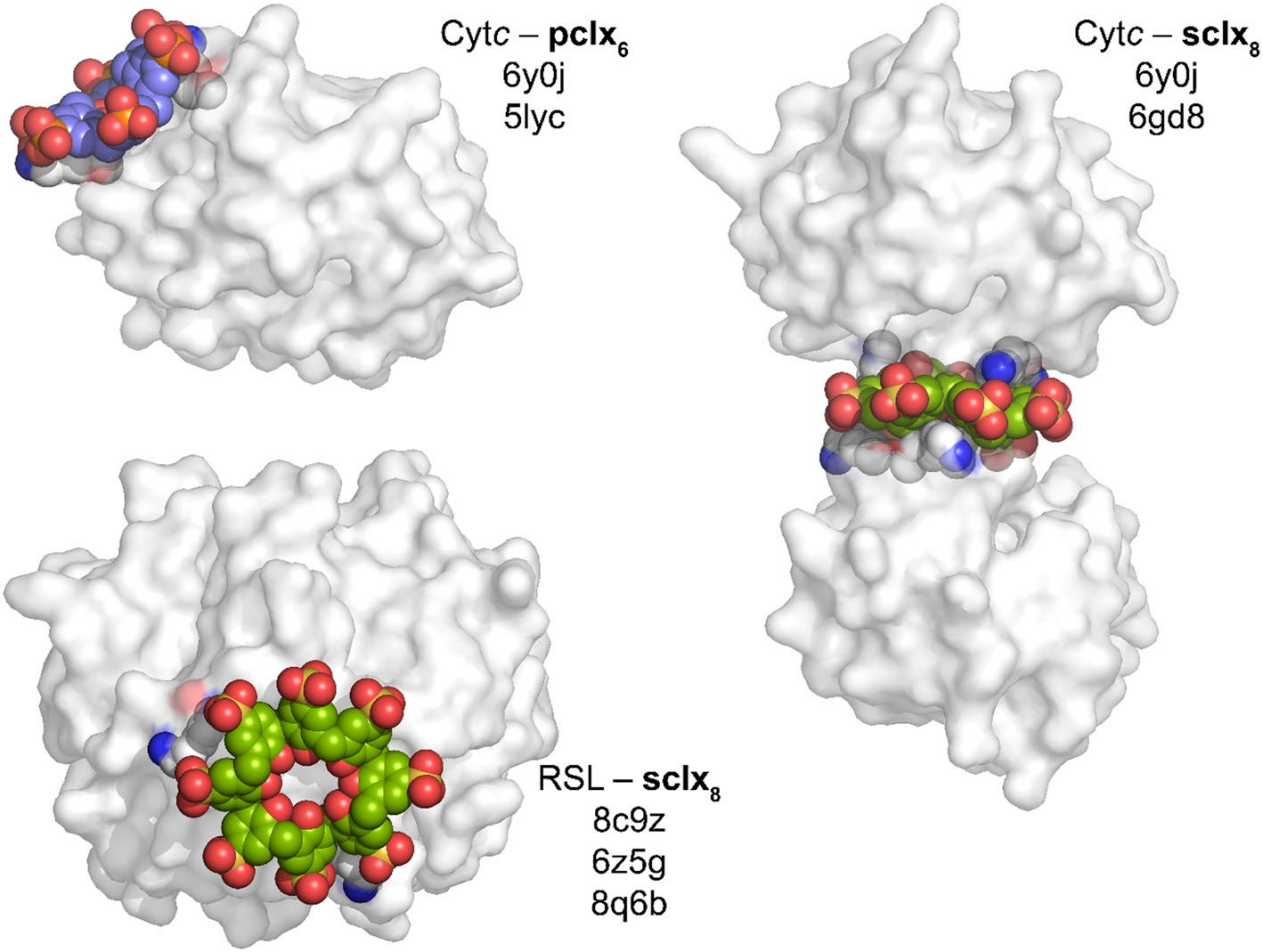
Examples of reproducible protein–calixarene interfaces (synthons). Distinct crystal structures featuring each synthon are indicated by representative PDB codes

A library of protein–calixarene cocrystal structures is in development (Crowley 2022), including different calixarenes with cytochrome *c* (p*I* ~ 9.5) (McGovern et al. 2012; Rennie et al. 2017, 2018; Doolan et al. 2018; Alex et al. 2018, 2020; Engilberge et al. 2019a, b; Mockler et al. 2021a, b; Flood et al. 2022), hen egg white lysozyme (HEWL, p*I* ~ 9.3) (McGovern et al. 2014a, 2014b), *Penicillium* antifungal protein (PAF, p*I* ~ 9.0) (Alex et al. 2019), a designed pentameric lectin (Pent, p*I* ~ 8.0) (Flood et al. 2024a) and *Ralstonia solanacearum* lectin (RSL, p*I* ∼6.5) (Ramberg et al. 2021b; Mockler et al. 2023, 2025a, b; Flood et al. 2024b). Using this collection of structures, protein–calixarene and calixarene–calixarene supramolecular synthons can be identified and applied to engineer new frameworks (Figs. 5 and 6, Table 1).

While the bowl-shaped calix[4]arene binds single residues on protein surfaces, the larger calix[6]arenes and calix[8]arenes can encapsulate two or more residues. Alex et al. (2019) reported the assembly of the ∼6 kDa and cationic *Penicillium* antifungal protein (PAF, p*I* ∼9.0) by **sclx**_**4**_, **sclx**_**6**_ or **sclx**_**8**_. Each calixarene acted as a molecular glue, yielding the first crystal structures of PAF (PDB 6ha4, 6hah and 6haj, resolution ≤ 1.50 Å). In all three PAF–**sclx**_**n**_ cocrystal structures, protein–calixarene interactions dominated the crystal packing, showcasing the *glue* capacity of **sclx**_**n**_. Notably, each calixarene bound at a similar patch on the protein surface, involving Pro29, Lys30 and Phe31. In the PAF–**sclx**_**6**_ structure, the calixarene adopted the 1,2,3-alternate double cone conformation, capturing Lys30 in a ‘cage’ comprising three phenolsulfonates and three phenolics (Fig. 5F). This binding mode, resembling the **sclx**_**4**_–lysine interaction, reoccurred in a cytochrome *c*–**sclx**_**6**_ cocrystal structure (PDB 6rgi) (Engilberge et al. 2019a). Recently, this supramolecular synthon was replicated using *p*-phosphonato-calix[6]arene (**pclx**_**6**_) in a cocrystal structure with MK-RSL (Mockler et al. 2025b).

**Fig. 7:**
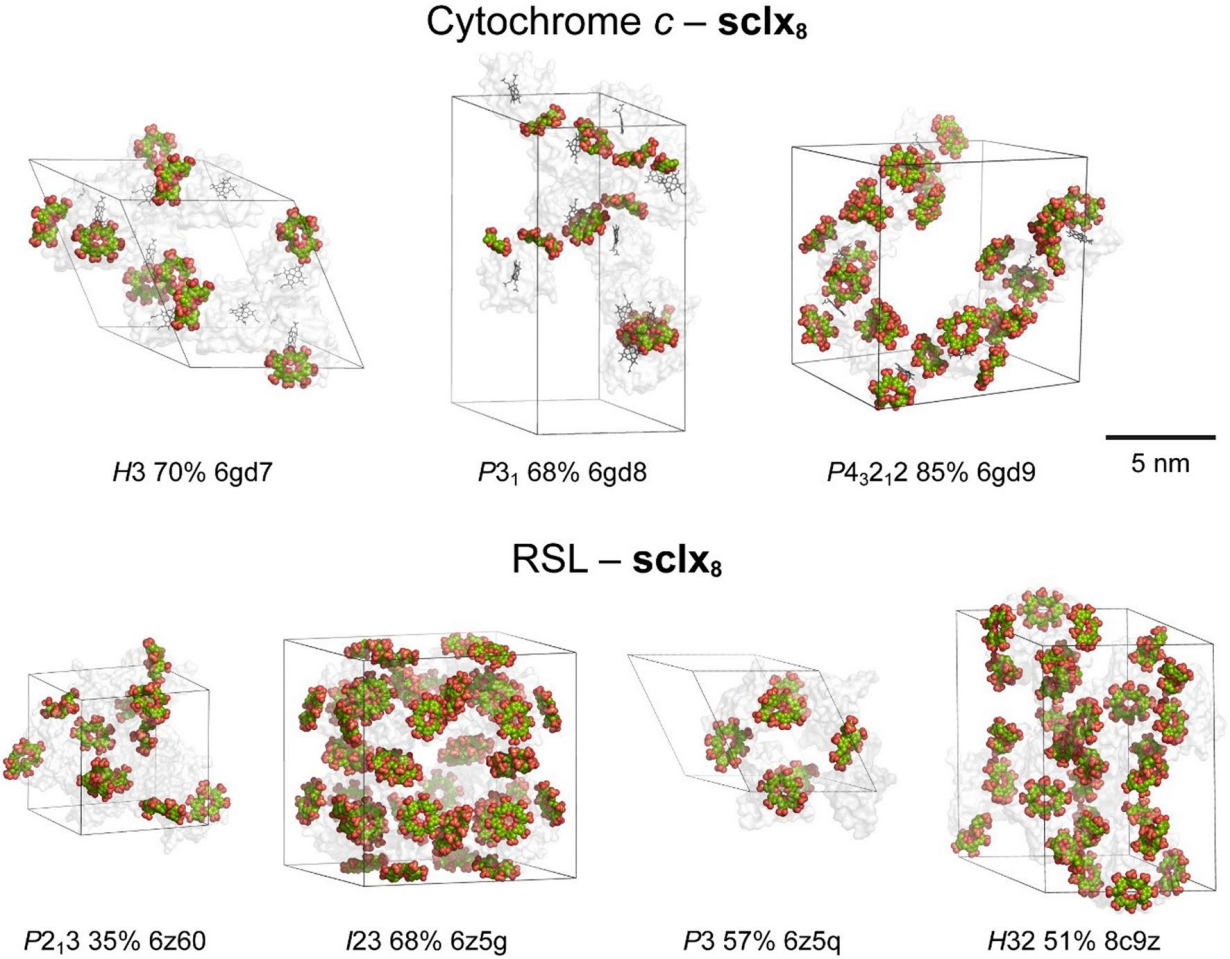
Protein–**sclx**_**8**_ polymorphs. Three cytochrome *c*–**sclx**_**8**_ frameworks and four RSL–**sclx**_**8**_ frameworks have been identified, with polymorph selection depending on the crystallization condition. Unit cells are shown, with space group, % solvent content and PDB codes indicated

Conformational flexibility increases with calixarene size affording a range of binding modes. The malleable **sclx**_**8**_ adopts multiple conformations (Perret et al. 2006), moulding to the protein surface and mediating different cocrystal forms (polymorphs) depending on the crystallization condition (pH and ionic strength) (Rennie et al. 2018; Engilberge et al. 2019b; Ramberg et al. 2021b; Mockler et al. 2023; Flood et al. 2024a). Protein–calixarene polymorphs arise when the protein and macrocycle tectons assemble via different supramolecular synthons (Mockler et al. 2023). This formation of several condition-dependent polymorphs with minimal building blocks offers potential for efficient materials fabrication (Fig. 7). For example, Rennie et al. (2018) reported three porous frameworks of cytochrome *c* with **sclx**_**8**_ in space groups *H*3 (PDB 6gd7), *P*3_1_ (PDB 6gd8) and *P*4_3_2_1_2 (PDB 6gd9). The latter framework is the most porous (85% solvent content, 5.6 nm pore diameters) and mediated entirely by protein–calixarene contacts. Later, Ramberg et al. (2021b) and Mockler et al. (2023) reported four crystalline frameworks of the ‘neutral’ *Ralstonia solanacearum* lectin (RSL, p*I* ∼6.5) mediated by **sclx**_**8**_. Three porous frameworks are obtained at pH ≤ 6 where RSL is cationic, favouring Coulombic attraction with the calixarene. These frameworks are directed exclusively by the calixarene, with no protein–protein contacts. In all four structures, the calixarene binds at a patch on the RSL surface involving Val13/Lys34, albeit with different calixarene conformations and binding modes. Another reproducible binding feature occurs at the Lys25/Lys83 patch, where **sclx**_**8**_ adopts the pleated loop conformation and masks ∼350 Å^2^ of the protein surface (Fig. 6). This RSL–**sclx**_**8**_ interface (synthon) reoccurs in three crystal structures with monomeric (PDB 8c9z) (Mockler et al. 2023), dimeric (PDB 6z5g) (Ramberg et al. 2021b) or trimeric (RSL-D32N mutant, PDB 8q6b) (Flood et al. 2024b) arrangements of the calixarene mediating the crystal packing.

The accumulating data suggests that predictable self-assembly of macrocycles, combined with specific protein recognition, affords a relatively simple strategy for engineering porous protein frameworks with defined dimensions.

## Macrocycle self-assembly: macrocycle–macrocycle synthons

An important aspect of the molecular glue capacity of macrocycles is their propensity for self-assembly (Talukdar et al. 2023). Given the protein recognition properties of macrocycles, predictable macrocycle oligomerization may be a useful tool for controlled protein assembly and crystal engineering. An early example comes from Salunke and coworkers, who reported cocrystal structures of an anionic porphyrin with three lectins: ConA (PDB 1jn2) (Goel et al. 2001), Jacalin (PDB 1pxd) (Goel et al. 2004) and Peanut lectin (PDB 1rir) (Goel et al. 2005). All three porous frameworks were mediated by *π − π* stacking of *meso*-tetrasulfonato-phenyl porphyrin. The porphyrin cores were assembled either *face-on* (H-aggregate) or *staggered* (J-aggregate) in the distinct structures. ConA assembly was mediated by a face-on porphyrin dimer while Jacalin assembly involved a staggered porphyrin dimer. The Peanut lectin framework was directed by staggered porphyrin trimers and tetramers.

**Fig. 8:**
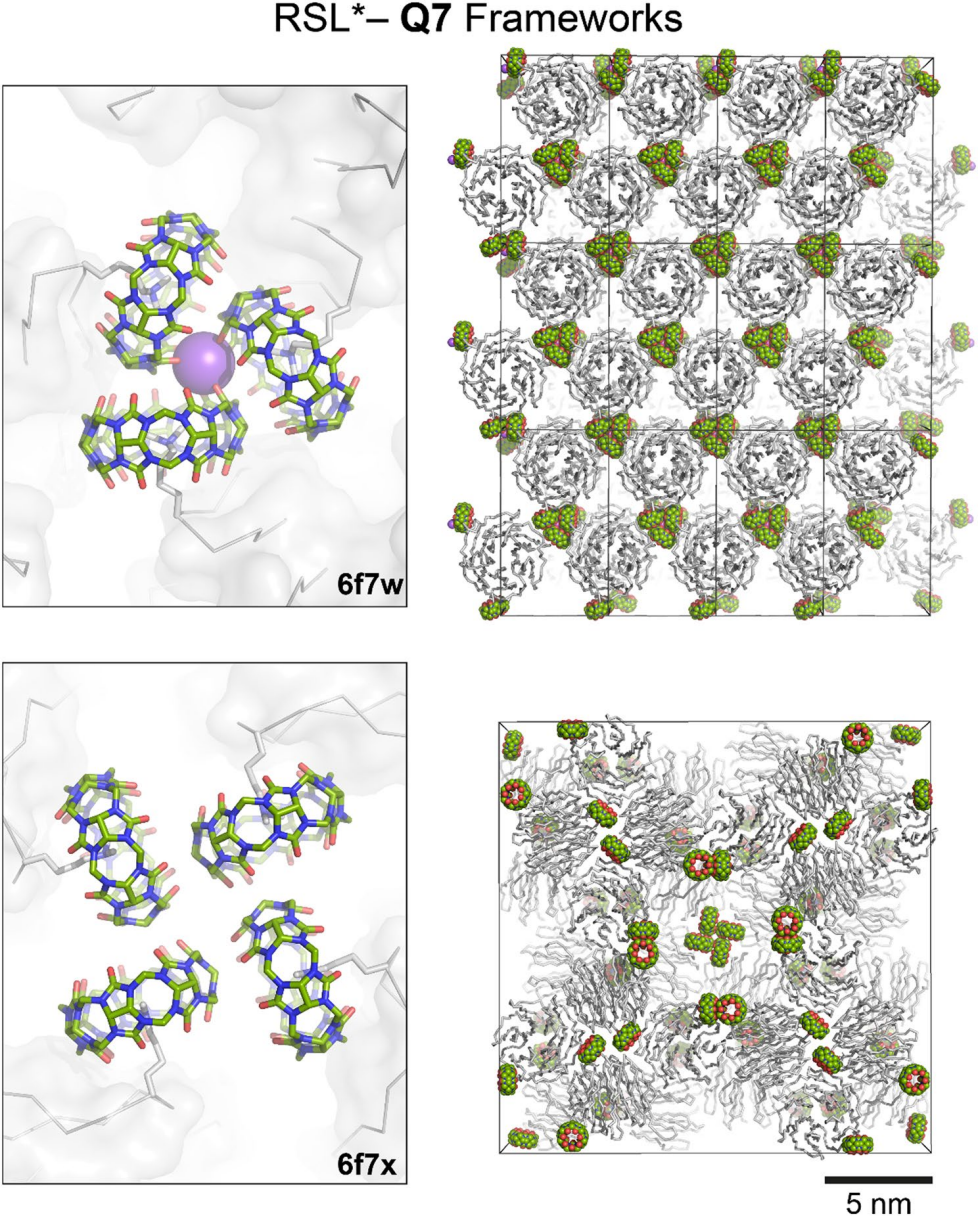
RSL*–**Q7** frameworks in space groups *C*222_1_ (PDB 6f7w, sheet assembly) and *F*432 (PDB 6f7x, cage assembly) mediated by cucurbituril clusters. Framework selection depends on the protein:**Q7** ratio and the sodium ion concentration of the crystallization condition

Likewise, cluster formation by cucurbiturils (Yang et al. 2020) appears to facilitate protein assembly (Guagnini et al. 2018, 2020a, b; Ramberg et al. 2021c; Ramberg and Crowley 2023). We reported nonterminal protein recognition by **Q7** on chemically dimethylated RSL (RSL*), with selective encapsulation of the most accessible dimethylated lysine (Lys34*) (Guagnini et al. 2018). Protein assembly within two crystal types was mediated by dimethyllysine recognition and **Q7** self-assembly. Trimeric or tetrameric cucurbituril clusters directed sheet (space group *C*222_1_, PDB 6f7w) or cage (space group *F*432, PDB 6f7x) assemblies of the protein, respectively, depending on the protein:**Q7** ratios and the sodium ion concentration (Fig. 8) (Guagnini et al. 2018; Ramberg and Crowley 2023). The sheet assembly (*C*222_1_) was amenable to crystal engineering (Guagnini et al. 2020b, 2020a; Ramberg et al. 2021c). For example, engineering an additional dimethylated lysine (Lys*) binding site on RSL (RSLex*) resulted in double the **Q7** binding capacity (Guagnini et al. 2020b). Later, Guagnini et al. (2020a) and Ramberg et al. (2021c) demonstrated that the protein layers could be modulated using metal binding (PDB 6zul) or by N-terminal coiled coil spacers (PDB 7p2j).

**Fig. 9:**
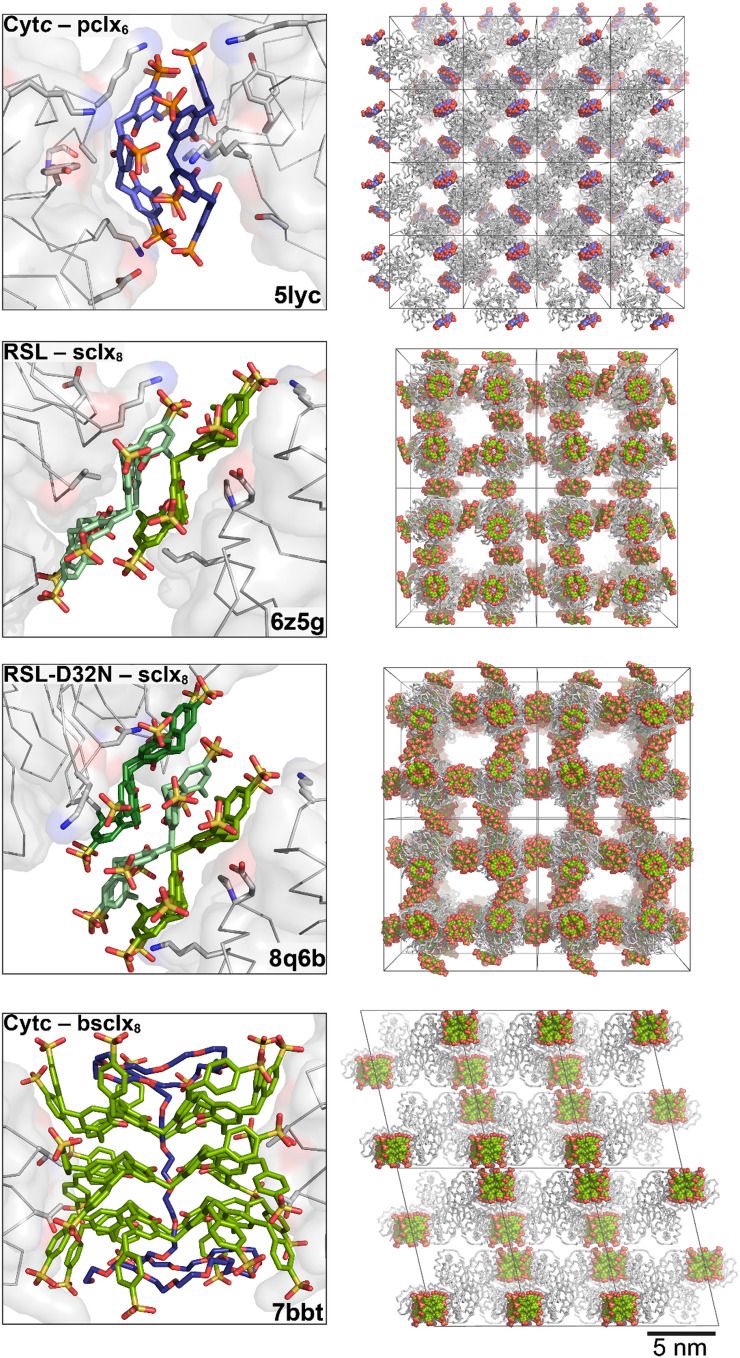
Examples of calixarene oligomers directing protein frameworks. Cytochrome *c*–**pclx**_**6**_, RSL–**sclx**_**8**_, RSL-D32N–**sclx**_**8**_ and cytochrome *c*–**b-sclx**_**8**_. PDB codes are indicated

Similar to the protein crystals mediated by π-stacked porphyrins or cucurbituril clusters, there are multiple examples of protein frameworks mediated by calixarene oligomers (Fig. 9). Rennie et al. (2017) reported a discus-shaped **pclx**_**6**_–**pclx**_**6**_ dimer that directed a *C*_2_-symmetric assembly of cytochrome *c* (PDB 5lyc, Fig. 9). Here, **pclx**_**6**_ adopted the double-cone conformation, and macrocycle dimerization was mediated by CH − *π* bonds, *π − π* bonds as well as hydrogen bonding. Each **pclx**_**6**_ monomer bound a patch on the cytochrome *c* surface involving two lysine residues, Lys4 and Lys100 (Fig. 9). Protein–**pclx**_**6**_, **pclx**_**6**_–**pclx**_**6**_ and protein–protein contacts yielded a porous assembly in space group *P*4_3_2_1_2 (62% solvent content, PDB 5 lyc). Later, we reported a ternary cocrystal of cytochrome *c, ***sclx**_**8**_ and **pclx**_**6**_, featuring the same dimeric **pclx**_**6**_ disc (Mockler et al. 2021a). Here, the two disparate calixarenes acted as alternate molecular glues to direct a porous, dendrite-like framework (73% solvent content, PDB 6y0j). Notably, the assembly was entirely composed of previously reported protein–protein, protein–calixarene and calixarene–calixarene interfaces (Rennie et al. 2017, 2018), highlighting the potential of calixarenes in protein crystal engineering. As observed in the binary cytochrome *c*–**pclx**_**6**_ structure (Rennie et al. 2017), **pclx**_**6**_ bound at the Lys4/Lys100 site (Fig. 6) and self-assembled to mediate a *C*_2_-symmetric cytochrome *c* dimer. Compared to the binary cocrystal, the relative orientation of the assembled proteins in the ternary cocrystal was altered by 180° rotation around the **pclx**_**6**_ dimer. A monomeric **sclx**_**8**_ bound at the Lys72/Lys73 patch and mediated another cytochrome *c* dimer, as observed in the *P*3_1_ cytochrome* c*–**sclx**_**8**_ crystal form (PDB 6gd8, Fig. 6) (Rennie et al. 2018). The distinct calixarenes acted as alternate linkers between protein nodes, forming supramolecular copolymers, which assembled via a known protein–protein interface (Rennie et al. 2017; Alex et al. 2018, 2020) forming the overall porous framework. Recently, we applied the dimeric **pclx**_**6**_ disc (synthon) to mediate RSL assembly (Mockler et al. 2025b).

**Fig. 10:**
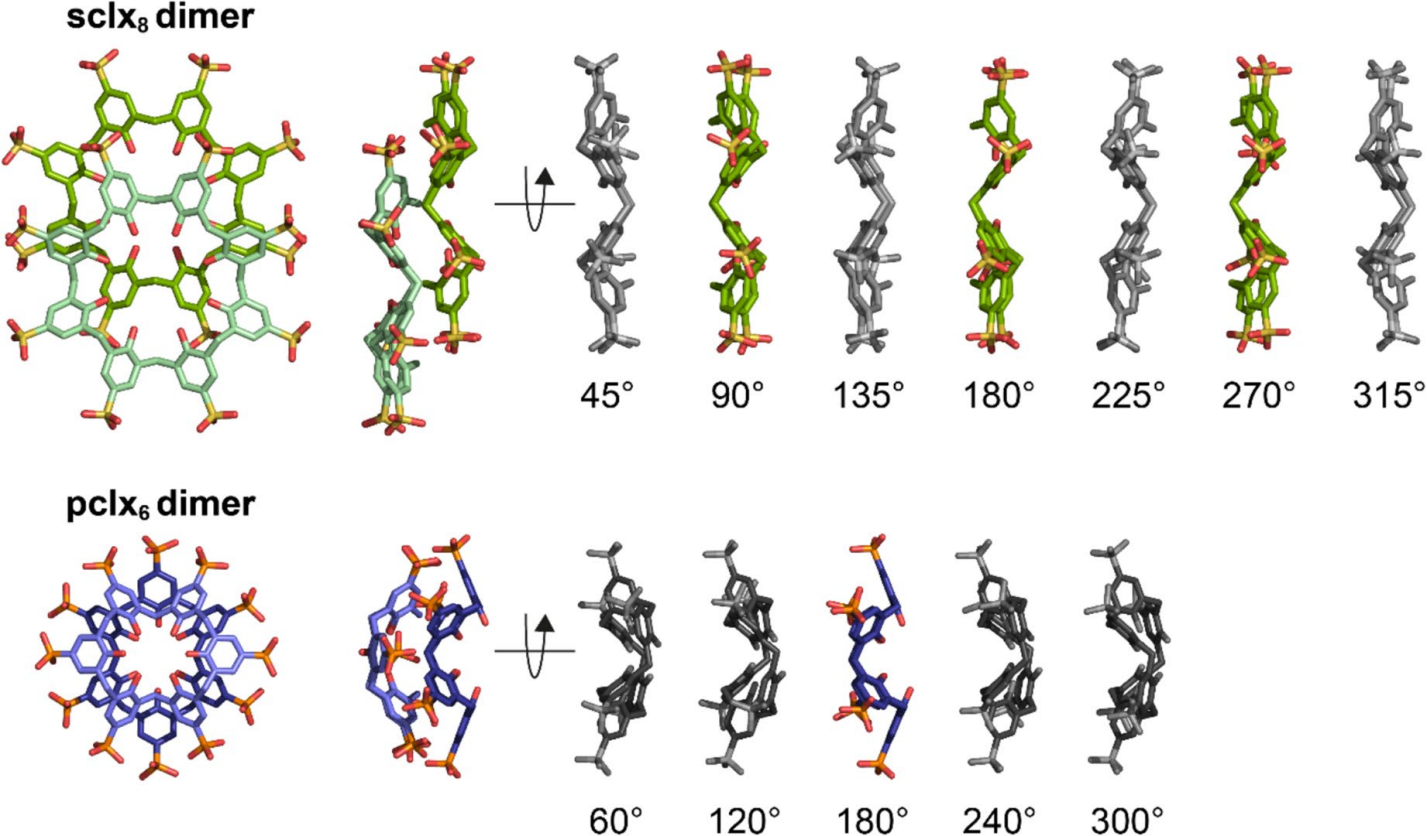
Dimeric calixarene synthons. **sclx**_**8**_ in the extended pleated loop conformation forms a staggered dimer. While **sclx**_**8**_ is *C*_8_-symmetric, the pleated loop conformation is *C*_4_-symmetric. Consequently, the **sclx**_**8**_–**sclx**_**8**_ synthon can form in four equivalent ways via 90° rotation of one monomer (green). In contrast, **pclx**_**6**_ adopts the *C*_2_-symmetric double-cone conformation and forms a face-on dimer in two equivalent ways via 180° rotation (blue)

**sclx**_**8**_ oligomers also mediate porous RSL frameworks (Ramberg et al. 2021b; Flood et al. 2024b). The most porous RSL–**sclx**_**8**_ polymorph is a cubic assembly (66% solvent content, space group *I*23) directed by staggered **sclx**_**8**_ dimers (Ramberg et al. 2021b). Each calixarene adopts the pleated loop conformation and assembles via CH − *π*, OH − *π*, *π − π* and anion − *π* interactions (PDB 6z5g, Fig. 9). This RSL–**sclx**_**8**_*I*23 form grows only at pH ≤ 4, favouring Coulombic attraction between the cationic protein and anionic macrocycle, as well as favouring macrocycle self-assembly. The pleated **sclx**_**8**_ can stack into dimers, trimers and higher order oligomers, as observed in two distinct protein–calixarene cocrystal structures as well as in the sodium–**sclx**_**8**_ salt. As such, the **sclx**_**8**_–**sclx**_**8**_ structural unit is a supramolecular synthon, with applications in protein crystal engineering. Flood et al. (2024b) reported assembly of an RSL mutant (RSL-D32N) mediated by staggered **sclx**_**8**_ trimers. An aspartic acid to asparagine mutation at one calixarene binding site resulted in a ∼10 Å shift of **sclx**_**8**_ across the protein surface. This shift, and the inclusion of RSL–**sclx**_**8**_* I*23 microseeds in the crystallization condition, together resulted in a porous (70% solvent content) framework directed by the **sclx**_**8**_ trimers. This ‘expanded’ form was also in cubic space group *I*23, with a 1.3 × increase in cell volume compared to the **sclx**_**8**_ dimer-mediated form (PDB 8q6b, Fig. 9). Rod-shaped crystals of the sodium–**sclx**_**8**_ salt grew in sodium citrate at pH 4–6, and the crystal structure revealed the same staggered stacks of the calixarene (CCDC 2298745). Based on these three structures and SAXS experiments, the conditions that favour this type of **sclx**_**8**_ oligomerization were identified as ∼3 M ionic strength, pH ∼4 (Ramberg et al. 2021b; Mockler et al. 2023; Flood et al. 2024b). The Na-**sclx**_**8**_ rods also grew at pH 5–6, but required high ionic strength (∼6 M) that may cause protein precipitation. This **sclx**_**8**_–**sclx**_**8**_ supramolecular synthon (Figs. 9 and 10) is useful for protein assembly, capable of directing porous protein frameworks.

A cocrystal structure of cytochrome* c* with an *extended-arm ***sclx**_**8**_ derivative, *p*-benzyl-sulfonato-calix[8]arene (**b-sclx**_**8**_, ∼2.2 kDa) revealed self-assembly of this large calixarene, but in a different way to **sclx**_**8**_ (Mockler et al. 2021b). The calixarene adopted the extended pleated loop conformation and stacked into cylindrical trimers, mediated by CH − *π* and *π − π* interactions. This calixarene stack presents four hydrophobic grooves, formed by the projected *extended-arms*, resulting in a unique protein-binding mode. Four cytochrome* c* molecules assembled around the calixarene stack by slotting the N-terminal α-helix into the hydrophobic groove. Surprisingly, a PEG fragment from the crystallization condition was threaded through the cavity of the calixarene stack, yielding a pseudorotaxane–protein cocrystal (PDB 7bbt, Fig. 9).

Identifying macrocycle self-assembly patterns has applications in protein crystal engineering, with protein-binding macrocycles acting as linkers between protein nodes. We are particularly interested in synthons that are transferable across different protein targets. These synthons include reproducible macrocycle–macrocycle and residue–macrocycle interfaces (including minimal binding tags) as discussed in the final sections.

## Modular assembly by calixarene dimers

The great variety of frameworks, such as MOFs, COFs, HOFs and POCs, arises in part from the modular ‘mix-and-match’ of exchangeable components (Eddaoudi et al. 2002; Jones et al. 2011; Furukawa et al. 2013; Feng et al. 2019; Geng et al. 2020; Little et al. 2020; Li et al. 2020). Over the last decade, similar concepts have aided protein crystal engineering (Sontz et al. 2015; Bailey et al. 2017; Partridge et al. 2021; Li et al. 2023). Macrocycles offer a versatile route to modular protein crystal engineering (Table 1). For example, calixarenes capable of predictable protein recognition and self-assembly may function as protein ‘linkers’. The RSL–**sclx**_**8**_ cubic (*I*23) framework inspired a modular ‘mix-and-match’ assembly in which the **sclx**_**8**_ dimer was swapped for the **pclx**_**6**_ discus dimer yielding a new framework (space group *H*32, PDB 9 hbd) with altered properties (Mockler et al. 2025b). The cubic RSL–**sclx**_**8**_ cocrystals grow only at pH 4, whereas the trigonal RSL–**pclx**_**6**_ cocrystals grow across a wide range of conditions and pH. Although **pclx**_**6**_ bound similar sites on RSL as **sclx**_**8**_, isoreticular frameworks such as those exemplified by MOFs are not possible due to the structural differences (size and shape) of the ‘interchangeable’ calixarene dimers. The disparate calixarene dimers directed different relative orientations of RSL, resulting in different packing and symmetries.

**Fig. 11:**
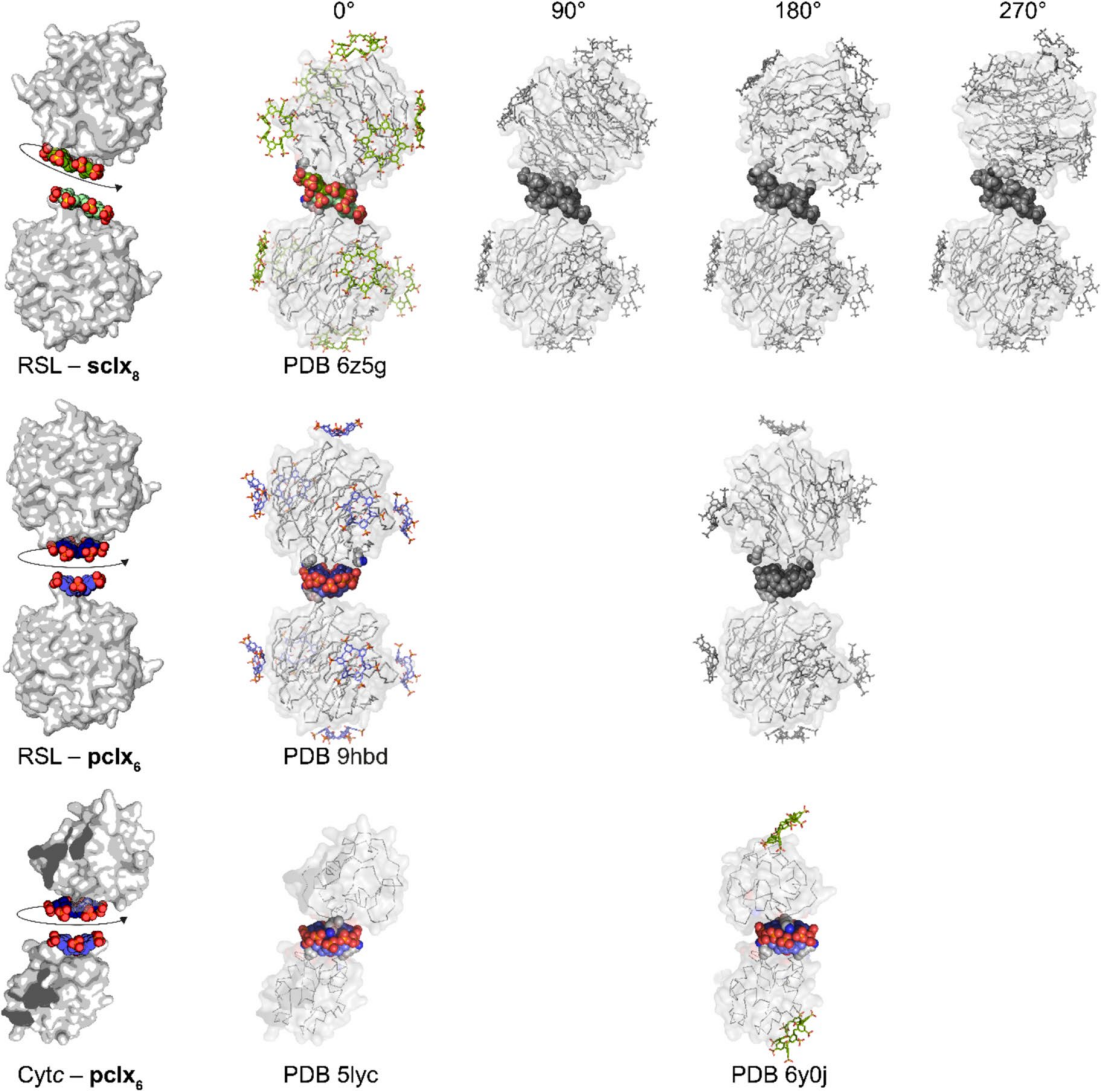
Crystal structures (PDB codes) and assembly models (grey). Models were built in COOT with two assumptions: (1) The protein–calixarene interfaces are fixed and (2) the calixarene–calixarene synthons occur as described in Fig. 10. The possible calixarene dimerization modes yield different relative orientations of the protein nodes. In the RSL case, only one of each assembly type has been identified in crystal structures (coloured models). The hypothetical assemblies (greyscale) have not been found. In the cytochrome *c* case, both assemblies have been identified in distinct crystal structures

In general, the calixarene (oligomer) is a linker that assembles protein nodes. The possible relative orientations of the protein nodes may vary depending on the calixarene dimerization mode. For instance, the **sclx**_**8**_–**sclx**_**8**_ synthon occurs in four equivalent ways via 90° rotation of one *C*_4_-symmetric calixarene monomer (Fig. 10). Therefore, assuming that the **sclx**_**8**_–protein-binding sites are fixed, the protein nodes can be assembled in four different relative orientations (Fig. 11). However, in the RSL–**sclx**_**8**_ case, only one of these orientations yields the cubic *I*23 structure (Ramberg et al. 2021b). This framework is mediated exclusively by the **sclx**_**8**_–**sclx**_**8**_ synthon, with no protein–protein interactions. Simple modelling revealed that this type of calixarene-mediated assembly is not possible with the alternate calixarene dimers (Fig. 11, greyscale), as packing gaps or clashes would occur. Contrasting with the **sclx**_**8**_ dimer, the **pclx**_**6**_–**pclx**_**6**_ synthon can occur in only two equivalent ways via 180° rotation, as a consequence of the *C*_2_-symmetric double-cone conformation (Fig. 10). Assuming the **pclx**_**6**_–protein-binding sites are fixed, two relative orientations of the protein nodes are possible (Fig. 11). This **pclx**_**6**_–**pclx**_**6**_ synthon mediated two types of cytochrome* c* assembly, with alternate relative orientations of the protein nodes in two distinct structures (Fig. 11) (Rennie et al. 2017; Mockler et al. 2021a). In the case of RSL–**pclx**_**6**_, only one of the possible assemblies has been identified (PDB 9hbd, Mockler et al. 2025b). Here, protein–protein contacts were required to assemble the calixarene-linked protein nodes. This result suggests that the macrocycle-mediated strategy could be integrated with computational interface design (Li et al. 2023) to produce predictable hierarchical frameworks. In the following section, we briefly discuss recent progress in simple protein engineering for controlled macrocycle binding.

## Protein engineering for controlled macrocycle binding

Ongoing research has identified the N-terminal methionine-lysine motif as a macrocycle binding site in solution (Hirani et al. 2018; Ramberg et al. 2021a). Recently, we provided crystallographic evidence of **sclx**_**4**_ or **pclx**_**6**_ binding at this engineered site on the model protein MK-RSL, suggesting the application of the N-terminal dipeptide motif as a macrocycle binding tag with consequences for protein assembly. While **sclx**_**4**_ is known to bind lysine and arginine residues (McGovern et al. 2012, 2014a; Alex et al. 2019), the MK-RSL–**sclx**_**4**_ crystal structure revealed a surprising result: the calixarene captured the N-terminal methionine (PDB 9gr3, Mockler et al. 2025a). Solution state NMR suggested **sclx**_**4**_ binding at either Met0 or Lys1. The latter binding mode was observed in MKA-RSL–**sclx**_**4**_ and MKAA-RSL–**sclx**_**4**_ cocrystal structures (PDB 9gr4 and 9gr5). By extending the N-terminal region with additional alanine residues, the steric accessibility of Lys1 was increased, resulting in **sclx**_**4**_ encapsulation via the familiar synthon (Fig. 5C). Each structure also included N-termini that were completely disordered and presumably not bound, highlighting the role of **sclx**_**4**_ in facilitating a disorder to order transition.

The N-terminal Met-Lys binding tag has also been applied in protein crystal engineering. Despite extensive screening across a wide range of conditions, a single RSL–**pclx**_**6**_ cocrystal form was mediated by the **pclx**_**6**_ dimer (space group *H*32, 44% solvent content, PDB 9 hbd) (Mockler et al. 2025b). Generating a new framework with RSL and **pclx**_**6**_ tectons required an additional feature for calixarene binding. The N-terminal Met-Lys motif modified calixarene binding, resulting in a porous cubic framework (space group *I*23, solvent content 53%, PDB 9hbf). In the crystal structure, **pclx**_**6**_ adopted the double cone conformation and bound selectively at the accessible Lys1 (Fig. 5F). To date, the N-terminal Met-Lys motif has enabled specific binding by **Q8** (Hirani et al. 2018), **Q6** (Ramberg et al. 2021a), **sclx**_**4**_ (Mockler et al. 2025a), **pclx**_**6**_ (Mockler et al. 2025b) and a sulfated terphenarene (Ifeagwu et al. 2025). Transferring this tag to other proteins may facilitate assembly and/or crystallization, with applications in both structural biology and materials fabrication. Furthermore, assemblies comprising different protein components may be attainable using this simple approach.

## Conclusions

While many different protein crystal engineering techniques are in development (Lanci et al. 2012; Sontz et al. 2015; Partridge et al. 2021; Li et al. 2023), we suggest that macrocycles are a particularly versatile tool. Using symmetric macrocycles may be an alternative to designing protein–protein interfaces that require extensive protein engineering. The homogenous macrocycle masks and thereby ‘simplifies’ a heterogeneous protein surface and provides a platform for assembly. Engineered assembly requires controlled macrocycle binding on the protein surface. Using a collection of crystal structures, predictable protein–calixarene and calixarene–calixarene binding motifs, i.e. supramolecular synthons, have been identified (Table 1). These synthons may be applied for crystal engineering with various proteins, natural or engineered. Modular crystal engineering, by the ‘mix-and-match’ of protein or calixarene components, is anticipated. Indeed, this method is relatively new, and the database of protein–calixarene cocrystal structures and synthons is limited (Table 1). Therefore, the possibilities that arise from protein–macrocycle cocrystallization require further exploration.

Calixarene-mediated protein assembly can facilitate crystallization, with applications in structural biology. For example, **sclx**_**n**_-mediated crystallization of PAF yielded the first crystal structures of the protein (Alex et al. 2019). Simple protein engineering can enable calixarene binding sites. For example, adding the N-terminal Met-Lys binding tag to RSL resulted in cocrystallization with **sclx**_**4**_ or **pclx**_**6**_, with the latter directing a porous framework. Minimal tags (1–3 residues) at protein termini are effective as they are solvent exposed and sterically accessible, promoting macrocycle binding without compromising protein structure or function (Armstrong et al. 2024; Mockler et al. 2025a).

In conclusion, macrocycles are a valuable addition to the toolbox of the structural biologist and crystal engineer alike. In addition to facilitating protein crystallization, predictable macrocycle-mediated assembly offers promising applications in the design of functional materials. We anticipate that the porosity, stability and functionality of such materials may be tuneable via component choice (e.g. calixarene dimers). As such, macrocycles may evolve from simple molecular glues into mediators of modular protein crystals with tailored properties. Current progress in protein design may further accelerate/complement this technique, enabling predictable macrocycle binding with minimal protein engineering.

## Data Availability

No datasets were generated or analysed during the current study.
